# Are Human Intestinal Eukaryotes Beneficial or Commensals?

**DOI:** 10.1371/journal.ppat.1005039

**Published:** 2015-08-13

**Authors:** Julius Lukeš, Christen Rune Stensvold, Kateřina Jirků-Pomajbíková, Laura Wegener Parfrey

**Affiliations:** 1 Institute of Parasitology, Biology Centre, České Budějovice, Czech Republic; 2 Faculty of Science, University of South Bohemia, České Budějovice, Czech Republic; 3 Canadian Institute for Advanced Research, Toronto, Canada; 4 Statens Serum Institut, Copenhagen, Denmark; 5 Departments of Botany and Zoology, University of British Columbia, Vancouver, Canada; University of Wisconsin Medical School, UNITED STATES

## Multiple Biomes (Virome, Microbiome, and Eukaryome) Govern Human Health

Since the advent of microbiology, it has been well known that each human body hosts a multitude of microbes. The magnitude of our microbial system is best reflected by the widely discussed ratio of one human cell to ten microbes. Indeed, humans and other mammals live in a consortium composed of vast arrays of viruses (these are typically called the virome), archaea and bacteria (i.e., the microbiome), along with fungi and other uni- and multicellular eukaryotes (protists and helminths, respectively) historically thought of as “parasites.” It was the advent of next generation sequencing (NGS) that first allowed deeper insight not only into the composition of this “microbial zoo” but also its dynamics in relation to age, diet, health, sex, and geographic location of the host. Attention has focused primarily on the bacterial microbiome, which constitutes the most abundant and diverse segment of the human intestinal ecosystem. However, we argue that eukaryotes play important, but largely unrecognized roles and that there is much to gain by turning our attention to eukaryotic members of the gut ecosystem.

## The Eukaryome Is Primarily Commensal

Diverse eukaryotes inhabit the human gut, including protists, fungi, and helminths [1,2], and we suggest the designation “eukaryome” for this collection, which appears more practical than the previously suggested “eukaryotome” [3]. Historically, any protist or helminth species found in humans was considered a parasite and assumed to have a pathogenic effect on the host organism [2]. Pathogenicity is certainly the rule for some intestinal protists, such as *Cryptosporidium* spp. and *Entamoeba histolytica*, as well as for the helminths *Ascaris lumbricoides* and *Strongyloides stercoralis*, especially in heavy infections or in malnourished or immunocompromised hosts [4]. However, critical evaluation of the literature indicates that for a range of human-associated protists and helminths, unambiguous data about their pathogenicity are hard to find (for a review, see [5]).

**Fig 1:**
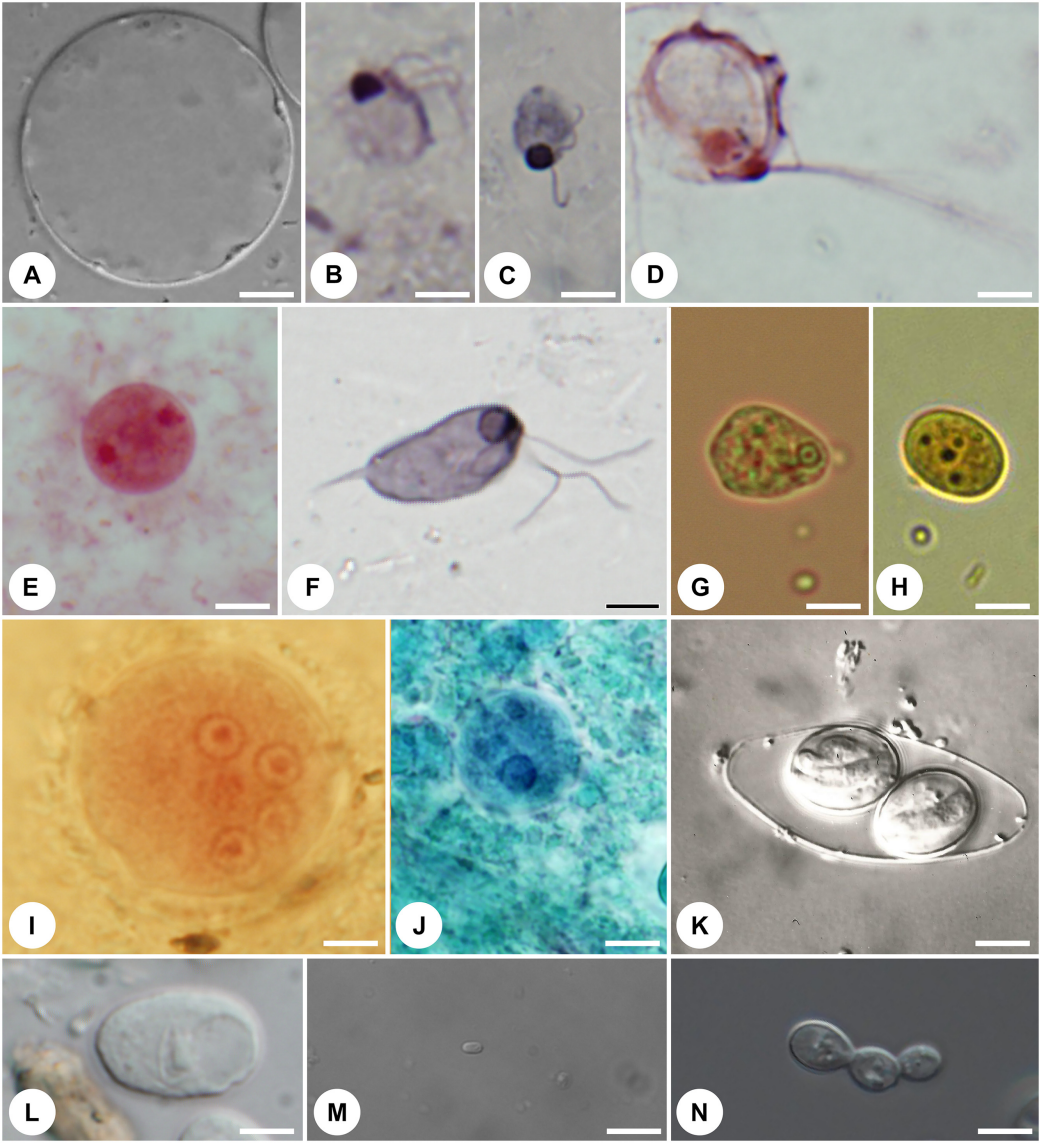
Representative protist symbionts that are part of the human gut eukaryome: (A) Stramenopile *Blastocystis hominis*; (B) diplomonadid *Enteromonas hominis*; (C) retortamonadid *Retortamonas intestinalis*; (D) trichomonadid *Pentratrichomonas hominis*; (E) tritrichomonadid *Dientamoeba fragilis*; (F) retortamonadid *Chilomastix mesnili*; (G) amoebozoans *Entamoeba hartmani*; (H) *Endolimax nana*; (I) *Entamoeba coli*; (J) *Entamoeba dispar*; (K) coccidian *Isospora belli*; (L) amoebozoan *Iodamoeba buetschli*; (M) microsporidian *Encephalitozoon cuniculi*; (N) ascomycete *Candida albicans*. Magnification is to the same scale (scale bar = 5 μm). Living cells (A,J–M), protargol-stained cells (B–D,F), trichrome-stained cell (E), Giemsa-stained cell (G), iodine-stained cell (H), and Gomori trichrome-stained cell (I). Pictures were kindly provided by K. Jirků-Pomajbíková (A,I,L), Ivan Čepička (B,C,E,F) (Charles University, Prague), Jaroslav Kulda (D) (Charles University, Prague), Marianne Lebbad (G,H) (Public Health Agency, Solna, Sweden), Martin Kostka (I) (University of South Bohemia, České Budějovice), Jiří Vávra (K) (Charles University, Prague), Martin Kváč and Bohouš Sak (M) (Institute of Parasitology, České Budějovice), and Miroslav Kolařík (N) (Institute of Microbiology, Prague).

Emerging evidence suggests that many common eukaryotic residents of the human gut are commensal or beneficial rather than parasitic, at least in some contexts. For example, the gut protist *Blastocystis* (Fig 1A) frequently associated in the literature with gastrointestinal disease, is very common, present in 10% to 100% of surveyed individuals (for a review, see [6]). Interestingly, several recent studies have found *Blastocystis* frequently in healthy individuals [7], sometimes at higher prevalence than in those with gastrointestinal disease [8]. Moreover, available data suggest that another protist, *Dientamoeba fragilis* (Fig 1E), has a similarly variable ecological role in the human intestinal ecosystem, occasionally associated with disease [9] but also highly prevalent in healthy individuals [7,8].

What differentiates pathogenic eukaryotes (true parasites) from commensals and mutualists? Many questions remain, but organismal factors such as duration of infection and/or colonization and localization within the gut are likely important. Organisms that cause acute disease and provoke an immune response that results in rapid clearance and long-term immunity (e.g., most Microsporidia (Fig 1M), *Cryptosporidium* spp., and *Giardia intestinalis*) are true parasites. At the other end of the spectrum, we find the beneficial eukaryotes that establish life-long associations with their host, including the cellulose-digesting ciliates and flagellates in herbivores [1]. In between, there are many eukaryotic organisms that colonize the intestine stably over long time periods and appear to be well tolerated by the immune system, including *Entamoeba coli*, *Blastocystis*, and *Dientamoeba*. Localization within the gut is also important. True parasites, such as *Entamoeba histolytica*, invade host tissue and migrate out of the gut, causing lesions, abscesses, and inflammation within the gut and throughout the body. In contrast, a commensal relationship is more likely for those organisms confined to the lumen of the gut, as exemplified by *Escherichia coli*, *Blastocystis*, *Dientamoeba*, *Enteromonas hominis* (Fig 1B), and *Retortamonas intestinalis* (Fig 1C). Moreover, the nature of the host–eukaryote relationship will change depending on the context of the host and the gut ecosystem, so that the same organism may be parasitic in some cases and commensal in others. The nutritional status and health of the host are clearly important in this equation. For example, symbionts will have a different effect on healthy humans with permanent access to food rich in vitamins and trace elements (as is common for citizens of industrialized countries) compared with malnourished or immunocompromised individuals.

Is the eukaryome beneficial overall? We do not know, and clear-cut cases of beneficial eukaryotes in the human gut are few. Yet, new findings in diverse fields suggest that we may ignore possible beneficial roles of the eukaryome at our peril. Potential benefits of the eukaryome may stem from increasing diversity in general or from the ability of eukaryotes to modulate the host immune system—each of which can induce changes in host physiology and the overall gut ecosystem.

## Diversity of the Eukaryome and the Gut Ecosystem

Diversity of the eukaryome as a whole may be beneficial. High diversity across all components of the gut ecosystem, including the eukaryome, is associated with healthy individuals and lower incidence of autoimmune and inflammatory disease [10–12]. Eukaryotes in the gut may signal, or even stimulate, higher diversity overall. Bacterial diversity is higher in the presence of *Entamoeba* (Fig 1G, 1I, and 1J) and some nematodes in the gut [13,14], but others did not see that effect [15,16]. However, eukaryome diversity is substantially lower in industrialized populations compared to populations with traditional lifestyles [7], just as it has been shown for bacteria [12,17]. This is a result of lifestyle changes (e.g., increased hygiene), as well as targeted removal. As mentioned above, most eukaryotes found in our body have been historically defined as parasites and assumed to have a negative impact. As such, doctors are (almost invariably) trained to remove them, even from asymptomatic patients [18,19]. Efficient drugs that disrupt their life cycles stand behind the virtual elimination of most protists and helminths from the populations of industrialized countries [20], considered a success of their medical systems. However, the true picture is possibly much more complicated, especially as eliminating eukaryotes is associated with increased incidence of immune-mediated and inflammatory diseases [10,11,20,21]. Indeed, the fast increase of the autoimmune and inflammatory diseases including Crohn’s disease, ulcerative colitis, various allergies, rheumatic arthritis, and others, are firmly associated with changes in the gut microbiome [11,12,22]. Amidst these changes, we propose that the overall rareness or even absence of the gut eukaryotes contributes significantly to this negative development.

## Immune System Modulation

Specific eukaryotes in the gut ecosystem can also have beneficial effects stemming from their ability to modulate the host immune system, which is well established for helminths. A positive role for helminths in the eukaryome is suggested by the negative correlation between their presence and incidence of immune-mediated disease and by studies documenting a rise in disease symptoms after clearance of parasites [10,20]. Indeed, direct introduction of helminths (helminth therapy) as a prophylactic or therapeutic agent has often been successful at preventing or treating autoimmune and inflammatory disease [10,11,20,21], with important counter examples. While currently unexplored, possible immuno-modulatory roles for *Blastocystis*, *Dientamoeba*, and other common intestinal protists that have co-evolved with humans are worth investigating thoroughly. One mechanism of action is the stimulation of mucus production, via the cytokine IL-22, which alleviates symptoms of colitis, improving gut health [23]. These sorts of indirect effects may be more common than currently appreciated because we have not looked for them. Perhaps *Blastocystis* is more common in healthy people because it helps maintain a healthy mucus layer in the intestine, either directly or through interactions with beneficial bacteria or the immune system.

## Rethinking the Term “Parasite”

In light of our evolving understanding of eukaryotes associated with humans and other hosts, we suggest referring to them as symbionts rather than parasites to capture the diversity of their ecological roles. The term “symbiont” encompasses all host-associated organisms across the spectrum of possible relationships (beneficial, detrimental, or neutral). This name change is part of the general shift away from viewing the eukaryome as inherently harmful, echoing the shift in perspective that already occurred for bacteria as we learned more about the vital and beneficial role of the bacterial microbiome in the human gut [24]. Many intestinal eukaryotes are commensal [2,5] or beneficial, for example, the ciliates and flagellates that break down cellulose (for a review, see [1]).

## Eukaryome and Its Relationships with Microbiome

Eukaryotic microbes co-evolved with mammals over millions of years and are a normal component of the microbiome from an evolutionary point of view [7,11]. Many are stable, long-term colonists rather than transient invaders [25]. The eukaryome can have strong effects on the composition and dynamics of the microbiome [14], likely with cascading consequences for our health. Although less numerous than bacteria, gut-dwelling eukaryotes are much bigger and they may have a disproportionate influence, similar to large animals in other ecosystems. For example, sharks on tropical reefs and wolves in Yellowstone have a profound effect on the entire ecosystem, and removal of these keystone species has wide consequences. It is worth testing whether targeted removal of eukaryotes—potential keystone components of the gut microbiome—in industrialized countries has contributed disproportionately to the diversity loss observed in the bacterial microbiome [12] and other negative health consequences discussed above. In summary, there are many exciting prospects for investigating potential benefits of the human eukaryome, all while keeping in mind the well-documented detrimental impact of some eukaryotic symbionts, particularly when present in large numbers and in mammalian hosts experiencing food limitation [26].

## Future Prospects

- Calling intestinal eukaryotic microbes symbionts—which encompasses mutualists, commensals, and parasites—rather than parasites conveys the diverse interactions they have with hosts above and beyond pathogenicity.
- There is a need to change therapeutic strategies to target elimination only of demonstrably pathogenic species—the true parasites—from host organisms, while avoiding removal of the harmless or commensal species.
- Now is the right time to determine the diversity of eukaryotic microbes in the healthy human population using NGS and to disentangle their relationships with bacterial communities.
- Characterizing eukaryotic microbes will enable us to associate the occurrence or absence of various eukaryotic microbes with human diseases.
- With the aim to bring some of the “lost” intestinal eukaryotes back, controlled colonization by commensal eukaryotes in human volunteers should be performed to confirm the predicted positive impact on diversification of human gut microbiome.
- If successful, these trials should be followed by controlled infections of patients with, e.g., functional and organic gastrointestinal diseases with a goal to inform novel approaches in the treatment of such diseases.
